# 76 Defining Social Prescribing Within a Physical Activity Context

**DOI:** 10.1093/eurpub/ckae114.157

**Published:** 2024-09-26

**Authors:** Rasmus Nielsen, Julie Sandell Jacobsen, Dea Kejlberg Andelius, Nanna Holt Jessen, Lene Gissel Rasmussen, Halfdan Thorsø Skjerning, Nina Sjørup Simonsen, Sebastian Skejø, Solvej Videbæk Bueno, Knud Ryom, Per Kallestrup, Jeanette Christensen

**Affiliations:** Aarhus University, Aarhus, Denmark; Research Unit for General Practice, Aarhus, Denmark; Research Unit for General Practice, Aarhus, Denmark; Research Centre for Health and Welfare Technology, Programme for Rehabilitation, VIA University College, Aarhus, Denmark; Aarhus University, Aarhus, Denmark; Research Unit for General Practice, Aarhus, Denmark; Research Unit for General Practice, Aarhus, Denmark; Aarhus University, Aarhus, Denmark; Research Unit for General Practice, Aarhus, Denmark; Research Unit for General Practice, Aarhus, Denmark; Research Unit for General Practice, Aarhus, Denmark; Aarhus University, Aarhus, Denmark; Research Unit for General Practice, Aarhus, Denmark; Aarhus University, Aarhus, Denmark; Research Unit for General Practice, Aarhus, Denmark; Aarhus University, Aarhus, Denmark; Research Unit for General Practice, Aarhus, Denmark; Research Unit for General Practice, Aarhus, Denmark; Research Unit of General Practice, Department of Public Health, University of Southern Denmark, Odense, Denmark; DRIVEN, Institute of Sports Science and Clinical Biomechanics, University of Southern Denmark, Odense, Denmark

## Abstract

**Purpose:**

Social prescribing involves linking citizens with non-medical services and support within their community to enhance their health and well-being. In social prescribing, referring citizens to physical activities are often emphasized, probably due to their already existing availability within most communities. Various definitions of social prescribing exist in the scientific literature. Since then, a call for constructive criticism and incorporation of unique perspectives from different contexts was made as it was acknowledged that the definition of social prescribing will evolve over time, including within the domain of physical activity. On this basis, the purpose of the present study was to develop a definition of social prescribing within a physical activity context.

**Methods:**

Based on previous consensus-based and non-consensus-based definitions of social prescribing published in scientific journals (n = 3), the authors (n = 12) of the present abstract co-produced a new definition of social prescribing within the context of physical activity based on face-to-face meetings and correspondence.

**Results:**

The new definition of social prescribing reads: “Social prescribing in a physical activity context has the overall goal of contributing to health and well-being through a process of trusted entities (e.g. GPs) referring individuals directly or through a link worker to groups that are physically active and that support social belongingness”. In this definition, the individual has medial or non-medical needs and is therefore in need of help from trusted entities. Trusted entities can be, but are not limited to: family, friends, health professionals, authorities, job-center personel and patient associations. Link workers are knowledgeable about the possibilities of physical activity in the local area and they support the individual in a co-creating manner to find the best fitting activity. Social belongingness is essential, meaning that the physical activity is not carried out on one’s own, but together with others, thereby supporting physical, mental and social well-being, including the need to belong.

**Conclusion:**

Given the rapid scaling and rollout of social prescribing globally, we hope this definition serves as a complementary contribution to the field of social prescribing by helping to build a common understanding of social prescribing within the field of physical activity.

